# Identification of the most specific markers to differentiate primary pulmonary carcinoma from metastatic gastrointestinal carcinoma to the lung

**DOI:** 10.1186/s13000-021-01184-2

**Published:** 2022-01-14

**Authors:** Bachar Alabdullah, Amir Hadji-Ashrafy

**Affiliations:** Department of Anatomical Pathology, University of Sydney, Nepean Hospital, Derby Street, Kingswood, NSW 2747 Australia

**Keywords:** Cancer. Lung. Gastrointestinal. Napsin-a. CK7. CK20. CDX2. TTF1. SATB2. Biomarkers

## Abstract

**Background:**

A number of biomarkers have the potential of differentiating between primary lung tumours and secondary lung tumours from the gastrointestinal tract, however, a standardised panel for that purpose does not exist yet. We aimed to identify the smallest panel that is most sensitive and specific at differentiating between primary lung tumours and secondary lung tumours from the gastrointestinal tract.

**Methods:**

A total of 170 samples were collected, including 140 primary and 30 non-primary lung tumours and staining for CK7, Napsin-A, TTF1, CK20, CDX2, and SATB2 was performed via tissue microarray. The data was then analysed using univariate regression models and a combination of multivariate regression models and Receiver Operating Characteristic (ROC) curves.

**Results:**

Univariate regression models confirmed the 6 biomarkers’ ability to independently predict the primary outcome (*p* < 0.001). Multivariate models of 2-biomarker combinations identified 11 combinations with statistically significant odds ratios (ORs) (*p* < 0.05), of which TTF1/CDX2 had the highest area under the curve (AUC) (0.983, 0.960–1.000 95% CI). The sensitivity, specificity, positive predictive value (PPV), and negative predictive value (NPV) were 75.7, 100, 100, and 37.5% respectively. Multivariate models of 3-biomarker combinations identified 4 combinations with statistically significant ORs (*p* < 0.05), of which CK7/CK20/SATB2 had the highest AUC (0.965, 0.930–1.000 95% CI). The sensitivity, specificity, PPV, and NPV were 85.1, 100, 100, and 41.7% respectively. Multivariate models of 4-biomarker combinations did not identify any combinations with statistically significant ORs (*p* < 0.05).

**Conclusions:**

The analysis identified the combination of CK7/CK20/SATB2 to be the smallest panel with the highest sensitivity (85.1%) and specificity (100%) for predicting tumour origin with an ROC AUC of 0.965 (*p* < 0.001; SE: 0.018, 0.930–1.000 95% CI).

**Supplementary Information:**

The online version contains supplementary material available at 10.1186/s13000-021-01184-2.

## Background

Lung cancer is the second most prevalent cancer in both men and women [1] and remains the leading cause of cancer-related deaths in both at 55.9 and 36.6 deaths per 100,000 respectively [1, 2]. The 2015 WHO Classification of Lung Tumors divides lung tumours into 6 main types and 77 subtypes based on histological appearance. The 6 main types are epithelial, neuroendocrine, mesenchymal, lymphohistocytic, tumours of ectopic origin, and metastatic tumours, while the most important subtypes include adenocarcinoma and squamous, small, and large-cell carcinomas [3]. Another method of classification looks at the neoplastic cells’ site of origin and classifies tumours as either primary (arising directly from the lungs), or secondary (metastasising to the lung from a distant site). The combination of the histological pattern and site of origin offers clinicians insight into the tumour’s staging, prognosis, and management options. Ensuring accurate identification and classification is of paramount importance in the ever growing age of immunotherapy, offering patients hope instead of the current grim outlook. Identification is traditionally done by correlating clinical evidence with radiological and pathological findings. Accurate classification falls on the shoulders of the anatomical pathologist and depends on a number of factors including the quality of the biopsy, the experience of the pathologist, and the extent of tumour differentiation. Well differentiated tumours have a clear histopathological pattern and are easy to classify. Poorly differentiated tumours on the other hand do not have a clear histopathological pattern and their classification was traditionally highly dependent on the pathologist’s level of expertise. This introduced inconsistencies in diagnosis, classification, and ultimately patient management. One emerging tool to assist with accurate identification and classification is the detection of specific biological markers (biomarkers) in tumour samples using immunohistochemical (IHC) staining techniques. Biomarkers are defined as “any substance, structure, or process that can be measured in the body or its products and influence or predict the incidence of outcome or disease” [4]. Some biomarkers are preferentially expressed in certain types of tissue but not in others, thus offering a method of objectively identifying the histological subtype of a tumour even if it exhibits poor differentiation.

Since their introduction, the use of biomarkers has become part of the routine diagnostic workup. Biomarkers traditionally used for the diagnosis of lung tumours include TTF1, Napsin-A, and CK7. Diagnosis of gastrointestinal tract (GIT) tumours requires a different set of biomarkers, the most useful of which include CK20, CDX2, and most recently SATB2. Despite recommendations for their use in aiding diagnosis, which biomarkers are used highly depends on the individual practices of institutions. TTF1 [5–7] and Napsin-A [5, 7] exhibit high sensitivity and specificity for well differentiated primary lung adenocarcinomas, but there are a number of reported cases of less differentiated samples that exhibit little to no staining with these two markers [7]. Moreover, there have been occasional cases of colonic adenocarcinomas that show strong and diffuse staining with TTF1 [7]. Similarly, there are reported cases of CK7 expression in primary GIT tumours [8]. CK20 is highly sensitive for the detection of colorectal carcinoma but has a low specificity [9]. CDX2 is preferentially expressed in the intestinal epithelium, thus is very sensitive for detecting colorectal adenocarcinoma [10, 11], but there have been reported cases of primary lung adenocarcinomas that express CDX2 [10, 11]. SATB2 has recently been shown to be highly sensitive for colorectal adenocarcinomas with sensitives that range between 80 and 97% [7, 9, 12–16]. Similar to its counterparts, SATB2 may still show low level expression in primary lung adenocarcinomas [12]. With all the shortcomings of the biomarkers currently used in practice, SATB2 has the potential of adding value in differentiating between primary and secondary lung tumours. Additionally, with the overabundance of markers that can be used for that purpose, there are no studies to date that attempt to identify the smallest, most optimal panel for differentiating primary and secondary lung tumours; this study attempts to do address that gap.

The study aims to investigate the differential expression of the biomarkers CK7, Napsin-A, TTF1, CK20, CDX2, and SATB2 in samples of primary lung, secondary lung, and primary GIT tumours to identify the smallest panel with the highest sensitivity and specificity for differentiating between primary lung tumours and metastatic GIT tumours to the lungs. Our main hypothesis states that the expression of CK7, Napsin-A, TTF1, CK20, CDX2, and SATB2 in lung tumours is dependent on the tumour’s site of origin. If true, we speculate that the use of these biomarkers will be significant in differentiating between primary and secondary lung tumours, with CK7, Napsin-A, and TTF1 being positive if primary, and CK20, CDX2, and SATB2 being positive if secondary. We also predict that a panel consisting of Napsin-A, TTF1, and SATB2 will be the most sensitive and specific at differentiating between primary and secondary lung tumours.

## Methods

A low-and-negligible risk (LNR) ethics application was prepared and approval from the Nepean and Blue Mountains Local Health District Human Research Ethics Committee was received on November 27th, 2017. Subsequently, a search was performed using a central directory of tumour biopsies and resections done at Nepean Hospital (NSW, Australia). The search looked at all lung resections and biopsies, and all GIT tumour resections done in the last 60 months. The search yielded 200 lung resections, 199 core lung biopsies, 7 wedge lung biopsies, and 29 colon tumour resections. Samples to be included in the study needed to be of a pattern common to both lung and GIT tumours (such as adenocarcinomas) to allow meaningful investigation of their origin. Hence, primary squamous and small-cell lung carcinomas were excluded. Secondary tumours not originating from the GIT (e.g. metastatic pancreatic adenocarcinomas) were also excluded as their biomarker staining pattern is different to that of GIT tumours. Subsequently, pathology reports were inspected and the case number, medical record number (MRN), name, age, sex, reported diagnosis, site of origin, specimen type, tumour block numbers, and previously done biomarker stain results were collected. The samples were then filtered and any duplicates (patients with more than one biopsy from the same site) were removed. The initial working set of 179 samples was then established and included 146 primary lung, 10 secondary lung, 21 primary GIT, and 2 undifferentiated tumours. Primary lung tumours included atypical and typical carcinoid tumours, neuroendocrine tumours, and pulmonary adenocarcinomas ranging from undifferentiated to well differentiated with variable subtypes including mucinous, non-mucinous adenocarcinoma in situ, acinar, lepidic, papillary, and micropapillary, Secondary lung tumours and primary GIT tumours were all adenocarcinomas originating from the colon and rectum. The 2 undifferentiated tumours were excluded from the analysis as they did not have an official classification. Of the 177 samples identified, 160 paraffin blocks were retrieved from storage and 17 were not found. These 17 were still included as their pathology reports showed previous biomarker stains that can be included in the analysis.

Each sample was then assigned a random number to facilitate blinding when interpreting results. This was done in excel by creating a column with a list of numbers from 1 to 177. The corresponding cells in the adjacent column were filled with randomly generated numbers using excel’s RAND function. Both columns were subsequently selected and the column containing the random numbers was sorted in ascending order, causing the first column to randomise, giving each sample a unique ID.

Staining of biomarkers was done using the tissue microarray method. This method creates individual slides with multiple, small tumour samples as opposed to the traditional method of having 1 tumour sample per slide. This allows for more rapid and cost efficient staining but increases the risk of unsuccessful stains. A punch size of 3 mm was utilised. Of the 160 blocks retrieved, 34 did not have sufficient volume to undergo tissue microarray. The 128 blocks left were melted and the appropriate number and volume of tissue was retrieved and re-paraffinised into new biomarker-specific blocks. Each new block included a control that is known to stain positive for the respective biomarker. In total, 13 CK7 (OV-TL, DAKO), 12 Napsin-A (MRQ60, CELLMARQUE), 8 TTF1 (SP141, VENTANA), 15 CK20 (KS, DAKO), 15 CDX2 (EPR2764Y, CELLMARQUE), and 15 SATB2 (EP281, CELLMARQUE) blocks were created. The new blocks were then used to make immunoperoxidase (IPX)-compatible slides for IHC staining using a Ventana machine. Following staining, the slides were interpreted independently by the chief and second investigators, and the results were recorded as either positive, negative, or unsuccessful. Any conflicts in interpretation were settled by consensus. Finally, the results of staining were added to the initial data set and samples with all 6 biomarkers missing were removed, leaving a total of 170 samples.

Logically, to obtain the smallest, most sensitive and specific panel of biomarkers for differentiating between primary and non-primary tumours (dichotomous outcome), binary logistic regression models that use ‘tumour origin’ as the dependent variable and the biomarkers as the covariates need to be created, starting with univariate models and moving on to multivariate models of 2 to 6 covariates. Firstly, the data was coded into SPSS (tumour origin: 0 = non-primary, 1 = primary; stains: 0 = negative, 1 = positive, 2 = missing). For our analysis, ‘non-primary’ included both secondary lung tumours and primary GIT tumours as both have the same staining pattern. The missing data points were computed into SPSS as to be excluded from the analysis. A univariate binary logistic regression analysis was performed with each of the 6 biomarkers as covariates [Table 1]. Subsequently, a multivariate binary logistic regression analysis was performed using 15 different 2-biomarker combinations [Table 2], 20 different 3-biomarker combinations [Table 3], and 15 different 4-biomarker combinations, and the predicted probability of each outcome was recorded as a new variable. The combinations with non-significant odds ratios (ORs) (*p* ≥ 0.05) were excluded from further analysis. Receiver Operating Characteristic (ROC) curves of the statistically significant (*p* < 0.05) combinations were constructed using the outcome’s ‘predicted probability’ as the test variable and the outcome (tumour origin) as the state variable. The area under the curve (AUC) was then used to compare models’ ability to predict the outcome [Tables 2 and 3].

**Table 1:** Outcomes of 6 different univariate binary logistic regression models for CK7, Napsin-A, TTF1, CK20, CDX2, and SATB2 showing the odds ratio (OR) and corresponding *p*-values where the OR signifies the odds of belonging to the ‘primary lung tumour’ group of the ‘tumour origin’ outcome.. The sensitivity, specificity, positive predictive value (PPV), and negative predictive value (NPV) of each biomarker when considered individually are also shown in relation to the detection of primary pulmonary carcinoma in the case of CK7, Napsin-A, and TTF-1 compared to primary gastrointestinal carcinoma for CK20, CDX2, and SATB2

#	Marker	OR	p-value	Sensitivity	Specificity	PPV	NPV
1	CK7	19.425	< 0.001	93.3%	58.3%	91.7%	63.6%
2	Napsin-A	30.083	< 0.001	76.0%	90.5%	97.9%	38.8%
3	TTF1	54.327	< 0.001	81.3%	92.6%	98.3%	49.0%
4	CK20	0.029	< 0.001	57.1%	96.3%	66.7%	94.6%
5	CDX2	0.003	< 0.001	94.1%	96.2%	80.0%	99.0%
6	SATB2	0.033	< 0.001	75.0%	90.9%	62.5%	94.7%

**Table 2:** Outcomes of binary logistic regression models of 15 different 2-biomarker combinations showing the odds ratio (OR) and the corresponding *p*-values of each biomarker when the other in the combination is controlled. A comparison of the area under the curve (AUC) and the corresponding *p*-values generated from the Receiver Operating Characteristic (ROC) curves for each of the 11 statistically significant 2-biomarker combinations is also shown. The OR here signifies the odds of belonging to the ‘primary lung tumour’ group of the ‘tumour origin’ outcome

#	Markers	OR	*p*-value	ROC AUC	*p*-value
1	CK7	1.6162 × 10^1^	< 0.001	0.877	< 0.001
	Napsin-A	1.5424 × 10^1^	0.001		
2	CK7	6.8660	0.005	0.893	< 0.001
	TTF1	2.6690 × 10^1^	< 0.001		
3	CK7	8.4970	0.012	0.848	< 0.001
	CK20	3.5000 × 10^−2^	< 0.001		
4	CK7	3.0468 × 10^8^	0.996	N/A	
	CDX2	2.7351 × 10^−10^	0.996		
5	CK7	1.8114 × 10^1^	< 0.001	0.883	< 0.001
	SATB2	7.9000 × 10^− 2^	0.001		
6	Napsin-A	3.7190	0.133	N/A	
	TTF1	6.1509 × 10^8^	0.996		
7	Napsin-A	9.2810	0.016	0.785	< 0.001
	CK20	5.1000 × 10^−2^	0.006		
8	Napsin-A	1.6645 × 10^1^	0.030	0.978	< 0.001
	CDX2	4.0000 × 10^−3^	< 0.001		
9	Napsin-A	2.5106 × 10^1^	0.001	0.943	< 0.001
	SATB2	2.3000 × 10^− 2^	< 0.001		
10	TTF1	3.5732 × 10^1^	0.002	0.935	< 0.001
	CK20	1.7000 × 10^− 2^	< 0.001		
11	TTF1	2.3587 × 10^1^	0.015	0.983	< 0.001
	CDX2	3.0000 × 10^− 3^	< 0.001		
12	TTF1	3.7699 × 10^1^	< 0.001	0.912	< 0.001
	SATB2	3.4000 × 10^−2^	< 0.001		
13	CK20	2.3810 × 10^−1^	0.268	N/A	
	CDX2	3.5179 × 10^− 10^	0.996		
14	CK20	7.0000 × 10^−3^	< 0.001	0.883	< 0.001
	SATB2	6.4000 × 10^−2^	0.004		
15	CDX2	2.0760 × 10^−10^	0.996	N/A	
	SATB2	4.6710 × 10^−9^	0.996

**Table 3:** Outcomes of binary logistic regression models of 20 different 3-biomarker combinations showing the odds ratio (OR) and the corresponding p-values of each biomarker when the others in the combination are controlled. A comparison of the area under the curve (AUC) and the corresponding *p*-values generated from the Receiver Operating Characteristic (ROC) curves for each of the 4 statistically significant 3-biomarker combinations is also shown. The OR here signifies the odds of belonging to the ‘primary lung tumour’ group of the ‘tumour origin’ outcome

#	Markers	OR	*p*-value	ROC AUC	*p*-value
1	TTF1	4.8484 × 10 [15]	0.994	N/A	
	CK20	1.0856 × 10^−17^	0.994		
	SATB2	2.5186 × 10^−9^	0.996		
2	TTF1	6.6786 × 10 [7]	0.994	N/A	
	CK20	3.7433 × 10^−8^	0.994		
	CDX2	9.0702 × 10^−16^	0.993		
3	TTF1	1.0066 × 10 [15]	0.994	N/A	
	CDX2	4.7399 × 10^− 24^	0.992		
	SATB2	4.2070 × 10^− 16^	0.994		
4	Napsin-A	9.5063 × 10 [6]	0.994	N/A	
	TTF1	4.2801 × 10^21^	0.992		
	SATB2	1.2550 × 10^−15^	0.993		
5	Napsin-A	7.5000 × 10^−1^	0.799	N/A	
	TTF1	2.2365 × 10 [15]	0.995		
	CK20	5.2321 × 10^− 9^	0.996		
6	Napsin-A	1.4559	0.823	N/A	
	TTF1	3.7603 × 10 [8]	0.996		
	CDX2	5.7080 × 10^−3^	0.000447		
7	Napsin-A	6.6000	0.078	N/A	
	CK20	0.0236	0.026		
	SATB2	0.0716	0.020		
8	Napsin-A	7.0029	0.189	N/A	
	CK20	0.7798	0.875		
	CDX2	4.3559 × 10^−10^	0.996		
9	Napsin-A	3.4919 × 10 [14]	0.993	N/A	
	CDX2	8.186 × 10^−23^	0.992		
	SATB2	5.3664 × 10^−15^	0.993		
10	CK7	8.2054	0.027	0.910	< 0.001
	TTF1	2.0773 × 10 [1]	0.002		
	SATB2	7.0791 × 10^−2^	0.005		
11	CK7	3.6169	0.201	N/A	
	TTF1	2.5458 × 10^1^	0.004		
	CK20	1.8967 × 10^−2^	0.001		
12	CK7	1.5920 × 10^8^	0.996	N/A	
	TTF1	1.1861 × 10^1^	0.063		
	CDX2	3.6135 × 10^−10^	0.996		
13	CK7	2.3990 × 10^1^	0.006	N/A	
	Napsin-A	2.0456	0.495		
	TTF1	8.2481 × 10^8^	0.996		
14	CK7	1.5328 × 10^1^	0.013	0.964	< 0.001
	Napsin-A	8.9019	0.031		
	SATB2	4.5187 × 10^− 2^	0.002		
15	CK7	1.1446 × 10^1^	0.024	0.808	0.004
	Napsin-A	7.5571	0.034		
	CK20	4.2770 × 10^−2^	0.007		
16	CK7	2.9564 × 10^8^	0.996	N/A	
	Napsin-A	8.3096	0.115		
	CDX2	4.4641 × 10^−10^	0.996		
17	CK7	3.3543 × 10^1^	0.005	0.965	< 0.001
	CK20	2.8180 × 10^−3^	0.000		
	SATB2	5.5332 × 10^−2^	0.021		
18	CK7	5.4692 × 10^7^	0.996	N/A	
	CK20	1.3333 × 10^−1^	0.158		
	CDX2	1.9131 × 10^−16^	0.994		
19	CK7	1.1369 × 10^8^	0.996	N/A	
	CDX2	8.8464 × 10^−17^	0.994		
	SATB2	1.5086 × 10^−8^	0.996		
20	CK20	7.634 × 10^−9^	0.997	N/A	
	CDX2	1.5376 × 10^−16^	0.994		
	SATB2	2.1357 × 10^− 8^	0.996		

## Results

Positive controls were observed on all slides indicating successful overall staining using the Ventana machine. However, staining of some individual samples within each slide was unsuccessful. Table 1 in additional file 1 summarises the proportion of successful stains for each biomarker.

Of the 143 samples stained for CK7, 119 (83.2%) were primary and 24 (16.8%) were non-primary. Of the 119 primary tumours, 111 (93.3%) stained positive and 8 (6.7%) stained negative. Of the 24 non-primary tumours, 14 (58.3%) stained negative and 10 (41.7%) stained positive [Table 2 in additional file 1].

Of the 146 samples stained for Napsin-A, 125 (85.6%) were primary and 21 (14.4%) were non-primary. Of the 125 primary tumours, 95 (76.0%) stained positive and 30 (24.0%) stained negative. Of the 21 non-primary tumours, 19 (90.5%) stained negative and 2 (9.5%) stained positive [Table 3 in additional file 1].

Of the 166 samples stained for TTF1, 139 (83.7%) were primary and 27 (16.3%) were non-primary. Of the 139 primary tumours, 113 (81.3%) stained positive and 26 (18.7%) stained negative. Of the 27 non-primary tumours, 25 (92.6%) stained negative and 2 (7.4%) stained positive [Table 4 in additional file 1].

Of the 123 samples stained for CK20, 109 (88.6%) were primary and 14 (11.4%) were non-primary. Of the 109 primary tumours, 105 (96.3%) stained negative and 4 (3.7%) stained positive. Of the 14 non-primary tumours, 8 (57.1%) stained positive and 6 (42.9%) stained negative [Table 5 in additional file 1].

Of the 121 samples stained for CDX2, 104 (86.0%) were primary and 17 (14.0%) were non-primary. Of the 104 primary tumours, 100 (96.2%) stained negative and 4 (3.8%) stained positive. Of the 17 non-primary tumours, 16 (94.1%) stained positive and 1 (5.9%) stained negative [Table 6 in additional file 1].

Of the 119 samples stained for SATB2, 99 (83.2%) were primary and 20 (16.8%) were non-primary. Of the 119 primary tumours, 90 (90.9%) stained negative and 9 (9.1%) stained positive. Of the 20 non-primary tumours, 15 (75.0%) stained positive and 5 (25.0%) stained negative [Table 7 in additional file 1].

Univariate binary logistic regression models showed a significant OR (*p* < 0.05) of 19.4, 30.1, 54.3, 0.029, 0.003, and 0.033 for CK7, Napsin-A, TTF1, CK20, CDX2, and SATB2 respectively. Table 1 also summarises the sensitivity, specificity, positive predictive value (PPV), and negative predictive value (NPV) of each marker.

Multivariate analysis was performed for each of the 15 2-biomarker combinations. Of the 15, 11 had statistically significant ORs (*p* < 0.05). ROC curves of the 11 combinations showed all 11 to have statistically significant (*p* < 0.05) AUC, with the combination of TTF1/CDX2 having the highest (0.983, 0.960–1.000 95% CI) [Table 2]. The sensitivity, specificity, PPV, and NPV of the TTF1/CDX2 panel were 75.7, 100, 100, and 37.5% respectively.

Multivariate analysis was performed for each of the 20 3-biomarker combinations. Of the 20, only 4 had statistically significant ORs (*p* < 0.05). ROC curves of the 4 combinations showed all 4 to have statistically significant (*p* < 0.05) AUC, with the combination of CK7/CK20/SATB2 having the highest (0.965, 0.930–1.000 95% CI) [Table 3]. The sensitivity, specificity, PPV, and NPV of the CK7/CK20/SATB2 panel were 85.1, 100, 100, and 41.7% respectively.

Multivariate analysis was performed for each of the 15 4-biomarker combinations, however, none of the combinations had statistically significant ORs (*p* < 0.05).

## Discussion

The results obtained from our investigation supports our main hypothesis of the dependency of biomarker expression on the tumour’s site of origin. Univariate analysis clearly showed that all 6 biomarkers were statistically significant at predicting the outcome [Table 1]. CK7, Napsin-A, and TTF1 all had ORs > 1, signifying that they are highly predictive of the outcome coded as 1 (primary origin). Of these 3, TTF1 had the largest OR at 54.3, which means that if a given lung cancer sample is positive for TTF1, the odds of it being primary are 54.3 times more than the odds of it being non-primary. CK20, CDX2, and SATB2 had ORs < 1, signifying that they are highly predictive of the outcome coded as 0 (non-primary origin). Of these 3, CDX2 had the smallest OR for outcome 1 at 0.003, thus reciprocally having the highest OR for outcome 0 at 333.3. This means that if a given lung cancer sample is positive for CDX2, the odds of it being non-primary are 333.3 times more than the odds of it being primary. With such high predictive abilities, the results appear promising at first, however, the flaws of each biomarker are appreciated upon inspection of the sensitivity, specificity, PPV, and NPV of each [Table 1]. For example, CK7 has a high sensitivity (93.3%) but a low specificity (58.3%). The high sensitivity indicates a low probability of a false negative result, hence a negative stain is likely to represent a true negative and can rule out primary origin. The low specificity signifies a high false positive rate, hence a positive stain is very likely to be false, hence cannot rule in primary origin. Napsin-A and TTF1 have the opposite problem of having a high specificity but a low sensitivity, thus are good at ruling in primary origin when positive but cannot rule out primary origin if negative. Similarly, CK20 and SATB2 have a high specificity but low sensitivity, thus are good at ruling in non-primary origin when positive (i.e. rule out primary origin), but cannot rule out non-primary origin (i.e. cannot rule in primary origin) when negative. CDX2 is the only biomarker that appears to have both a high sensitivity and specificity, however, it is also the marker associated with the highest standard error (SE) for its OR (reciprocal OR: 333.3, SE 1.150, 4.2–3816.8 95% CI).

Unlike univariate models, multivariate models have at least 2 ORs (1 per covariate), thus their ability to predict the outcome cannot be judged just by comparing the values of their ORs. Consequently, ROC curves were utilised for that purpose by plotting the model’s sensitivity on the y-axis and 1-specficity on the x-axis then computing the AUC. A model with a high sensitivity and a high specificity (i.e. low 1-specificity) will have an ROC curve that hugs the upper left corner, hence maximising the AUC. Of all the statistically significant multivariate models [Tables 2 and 3], the combinations of TTF1/CDX2 and CK7/CK20/SATB2 had the highest AUC (0.983 and 0.965) amongst their peers. This is contrary to our initial prediction of TTF1/Napsin-A/SATB2 being the optimal panel. The TTF1/CDX2 panel had a moderate sensitivity of 75.7%, very high specificity of 100%, and a PPV and NPV of 100 and 37.5% respectively when the positive test is defined as TTF1+/CDX2-. When compared to TTF1 or CDX2 individually, despite a slight improvement in specificity and PPV, the sensitivity and NPV decrease when compared to both (more drastically when compared to CDX2). Based on that, it is difficult to recommend the TTF1/CDX2 panel over either of its constituents individually. The CK7/CK20/SATB2 panel improves on TTF1/CDX2 slightly with 85.1% sensitivity, 100% specificity, and a PPV and NPV of 100 and 41.7% respectively when a positive test is defined as CK7+/CK20−/SATB2-. When compared to its individual constituents, it appears that the 3-biomarker panel overcomes some of the individual flaws discussed earlier. The reasonably high sensitivity overcomes the low to moderate sensitivities of CK20 and SATB2, and the high specificity overcomes the low specificity of CK7. The NPV of the panel, however, is much lower than that of its constituents, but despite that, it still appears that the CK7/CK20/SATB2 panel is superior to both the individual constituents as well as the TTF1/CDX2 panel, hence can be recommended over them. None of the 4-biomarker combinations were statistically significant, hence analysis stopped at 3-biomakers.

## Conclusion

The data appears to confirm that panels with more than 3 biomarkers do not offer any additional value in differentiating between primary and non-primary tumour origin. Specifically, a panel made up of CK7, CK20, and SATB2 was shown to be the most sensitive and specific while overcoming the shortcomings of its individual constituents. We believe standardising testing to only include CK7, CK20, and SATB2 will not only provide the best quality of evidence in aiding diagnosis, but will also contribute to significant reduction in sample processing times and associated cost, hence should be adapted in all laboratories.

## Supplementary Information

**Additional file 1.**

## Data Availability

The data used and analysed are available from the corresponding author on request via email at bacharalabdullah@gmail.com.
