# Supplementary material for: Identification of the most specific markers to differentiate primary pulmonary carcinoma from metastatic gastrointestinal carcinoma to the lung
Source: Diagn Pathol. 2022 Jan 14;17:7. doi: 10.1186/s13000-021-01184-2 (PMC8759183; doi:10.1186/s13000-021-01184-2)
Supplement: Supplementary file 1 — Additional file 1. [file 13000_2021_1184_MOESM1_ESM.docx]

Table 1: Summary of the proportion of successfully stained samples in relation to the samples that were unsuccessful and the total number of samples in the set.

| Case Processing Summary | | | | | | |
| --- | --- | --- | --- | --- | --- | --- |
| Markers | Cases | | | | | |
|  | Valid | | Missing | | Total | |
|  | N | Percent | N | Percent | N | Percent |
| CK7 | 143 | 84.1% | 27 | 15.9% | 170 | 100.0% |
| Napsin-A | 146 | 85.9% | 24 | 14.1% | 170 | 100.0% |
| TTF1 | 166 | 97.6% | 4 | 2.4% | 170 | 100.0% |
| CK20 | 123 | 72.4% | 47 | 27.6% | 170 | 100.0% |
| CDX2 | 121 | 71.2% | 49 | 28.8% | 170 | 100.0% |
| SATB2 | 119 | 70.0% | 51 | 30.0% | 170 | 100.0% |

Table 2: Crosstabulation of CK7 vs. Tumour origin

|  | | | Tumour origin | | Total |
| --- | --- | --- | --- | --- | --- |
|  |  |  | Non-primary | Primary |  |
| CK7 | Negative | Count | 14 | 8 | 22 |
|  |  | % within CK7 | 63.6% | 36.4% | 100.0% |
|  |  | % within Tumour origin | 58.3% | 6.7% | 15.4% |
|  |  | % of Total | 9.8% | 5.6% | 15.4% |
|  | Positive | Count | 10 | 111 | 121 |
|  |  | % within CK7 | 8.3% | 91.7% | 100.0% |
|  |  | % within Tumour origin | 41.7% | 93.3% | 84.6% |
|  |  | % of Total | 7.0% | 77.6% | 84.6% |
| Total | | Count | 24 | 119 | 143 |
|  |  | % within CK7 | 16.8% | 83.2% | 100.0% |
|  |  | % within Tumour origin | 100.0% | 100.0% | 100.0% |
|  |  | % of Total | 16.8% | 83.2% | 100.0% |

Table 3: Crosstabulation of Napsin-A vs. Tumour origin

|  | | | Tumour origin | | Total |
| --- | --- | --- | --- | --- | --- |
|  |  |  | Non-primary | Primary |  |
| Napsin-A | Negative | Count | 19 | 30 | 49 |
|  |  | % within Napsin-A | 38.8% | 61.2% | 100.0% |
|  |  | % within Tumour origin | 90.5% | 24.0% | 33.6% |
|  |  | % of Total | 13.0% | 20.5% | 33.6% |
|  | Positive | Count | 2 | 95 | 97 |
|  |  | % within Napsin-A | 2.1% | 97.9% | 100.0% |
|  |  | % within Tumour origin | 9.5% | 76.0% | 66.4% |
|  |  | % of Total | 1.4% | 65.1% | 66.4% |
| Total | | Count | 21 | 125 | 146 |
|  |  | % within Napsin-A | 14.4% | 85.6% | 100.0% |
|  |  | % within Tumour origin | 100.0% | 100.0% | 100.0% |
|  |  | % of Total | 14.4% | 85.6% | 100.0% |

Table 4: Crosstabulation of TTF1 vs. Tumour origin

|  | | | Tumour origin | | Total |
| --- | --- | --- | --- | --- | --- |
|  |  |  | Non-primary | Primary |  |
| TTF1 | Negative | Count | 25 | 26 | 51 |
|  |  | % within TTF1 | 49.0% | 51.0% | 100.0% |
|  |  | % within Tumour origin | 92.6% | 18.7% | 30.7% |
|  |  | % of Total | 15.1% | 15.7% | 30.7% |
|  | Positive | Count | 2 | 113 | 115 |
|  |  | % within TTF1 | 1.7% | 98.3% | 100.0% |
|  |  | % within Tumour origin | 7.4% | 81.3% | 69.3% |
|  |  | % of Total | 1.2% | 68.1% | 69.3% |
| Total | | Count | 27 | 139 | 166 |
|  |  | % within TTF1 | 16.3% | 83.7% | 100.0% |
|  |  | % within Tumour origin | 100.0% | 100.0% | 100.0% |
|  |  | % of Total | 16.3% | 83.7% | 100.0% |

Table 5: Crosstabulation of CK20 vs. Tumour origin

|  | | | Tumour origin | | Total |
| --- | --- | --- | --- | --- | --- |
|  |  |  | Non-primary | Primary |  |
| CK20 | Negative | Count | 6 | 105 | 111 |
|  |  | % within CK20 | 5.4% | 94.6% | 100.0% |
|  |  | % within Tumour origin | 42.9% | 96.3% | 90.2% |
|  |  | % of Total | 4.9% | 85.4% | 90.2% |
|  | Positive | Count | 8 | 4 | 12 |
|  |  | % within CK20 | 66.7% | 33.3% | 100.0% |
|  |  | % within Tumour origin | 57.1% | 3.7% | 9.8% |
|  |  | % of Total | 6.5% | 3.3% | 9.8% |
| Total | | Count | 14 | 109 | 123 |
|  |  | % within CK20 | 11.4% | 88.6% | 100.0% |
|  |  | % within Tumour origin | 100.0% | 100.0% | 100.0% |
|  |  | % of Total | 11.4% | 88.6% | 100.0% |

Table 6: Crosstabulation of CDX2 vs. Tumour origin

|  | | | Tumour origin | | Total |
| --- | --- | --- | --- | --- | --- |
|  |  |  | Non-primary | Primary |  |
| CDX2 | Negative | Count | 1 | 100 | 101 |
|  |  | % within CDX2 | 1.0% | 99.0% | 100.0% |
|  |  | % within Tumour origin | 5.9% | 96.2% | 83.5% |
|  |  | % of Total | 0.8% | 82.6% | 83.5% |
|  | Positive | Count | 16 | 4 | 20 |
|  |  | % within CDX2 | 80.0% | 20.0% | 100.0% |
|  |  | % within Tumour origin | 94.1% | 3.8% | 16.5% |
|  |  | % of Total | 13.2% | 3.3% | 16.5% |
| Total | | Count | 17 | 104 | 121 |
|  |  | % within CDX2 | 14.0% | 86.0% | 100.0% |
|  |  | % within Tumour origin | 100.0% | 100.0% | 100.0% |
|  |  | % of Total | 14.0% | 86.0% | 100.0% |

Table 7: Crosstabulation of SATB2 vs. Tumour origin

|  | | | Tumour origin | | Total |
| --- | --- | --- | --- | --- | --- |
|  |  |  | Non-primary | Primary |  |
| SATB2 | Negative | Count | 5 | 90 | 95 |
|  |  | % within SATB2 | 5.3% | 94.7% | 100.0% |
|  |  | % within Tumour origin | 25.0% | 90.9% | 79.8% |
|  |  | % of Total | 4.2% | 75.6% | 79.8% |
|  | Positive | Count | 15 | 9 | 24 |
|  |  | % within SATB2 | 62.5% | 37.5% | 100.0% |
|  |  | % within Tumour origin | 75.0% | 9.1% | 20.2% |
|  |  | % of Total | 12.6% | 7.6% | 20.2% |
| Total | | Count | 20 | 99 | 119 |
|  |  | % within SATB2 | 16.8% | 83.2% | 100.0% |
|  |  | % within Tumour origin | 100.0% | 100.0% | 100.0% |
|  |  | % of Total | 16.8% | 83.2% | 100.0% |
